# Treatment of Tetanus

**Published:** 1912-06

**Authors:** R. H. L. Bibb


R. H. L. BIBB.

---

TREATMENT OF TETANUS.—In the writer's not altogether limited experience, Baccelli's, Rome, treatment is the only method that has given him other results than death in the treatment of tetanus. It was successful in all of four cases in whom he used it. It is cheap and is in easy reach of the most out-of-the-way practitioner, and may be administered without costly, complicated paraphernalia—an ordinary hypodermic syringe being all that is required. It consists in the subcutaneous injection—*not intravenous,* as recommended by Bernart, of Chicago—of a 2 or 3-per cent solution of carbolic acid in water. Begin—according to the severity of the case—with 5 to 8 drops of this solution twice or thrice daily, and gradually increase the quantity to 15 to 25 drops, as often, watching for the appearance of a greenish, smoky discoloration of the urine—the danger signal, a "bottle green" color. Until this discoloration appears, the remedy may be gradually increased with perfect safety—up to, according to Bernart, as much as 8 grains daily, but should be promptly reduced upon its appearance until only a faint outline of its remains. Tetanus seems to establish a sort of tolerance for carbolic acid as peritonitis does for opium; and there is as much, if not more, danger of giving too little of the remedy as there is in giving too much of it. Tetanus is a disease of short duration—my cases were all out of danger in ten days, and what is to be done must be done quickly. Carbolic acid injections do not preclude the use of other drugs that may be indicated—such as chloral, the bromides, etc.

According to Baccelli, the mortality of tetanus has been reduced to 2 or 3 per cent in mild, and to 18 per cent in severe cases wherever his method has been properly used.

R. H. L. BIBB.

---

The sooner a hollow bone is opened in acute osteomyelitis the less will be the destruction of bone.—*Am. Journal of Surgery.*

